# Effect of jute yarn on the mechanical behavior of concrete composites

**DOI:** 10.1186/s40064-015-1504-7

**Published:** 2015-11-25

**Authors:** Mohammad Zakaria, Mashud Ahmed, Md. Mozammel Hoque, Abdul Hannan

**Affiliations:** Dhaka University of Engineering and Technology, Gazipur, Bangladesh; Bangladesh University of Textiles, Tejgaon, Dhaka Bangladesh

## Abstract

The objective of the study is to investigate the effect of introducing jute yarn on the mechanical properties of concrete. Jute fibre is produced abundantly in Bangladesh and hence, very cheap. The investigation on the enhancement of mechanical properties of concrete with jute yarn as reinforcement, if enhanced, will not only explore a way to improve the properties of concrete, it will also explore the use of jute and restrict the utilization of polymer which is environmentally detrimental. To accomplish the objective, an experimental investigation of the compressive, flexural and tensile strengths of Jute Yarn Reinforced Concrete composites (JYRCC) has been conducted. Cylinders, prisms and cubes of standard dimensions have been made to introducing jute yarn varying the mix ratio of the ingredients in concrete, water cement ratio, length and volume of yarn to know the effect of parameters as mentioned. Compressive, flexural and tensile strength tests had been conducted on the prepared samples by appropriate testing apparatus following Standards of tests. Mechanical properties of JYRCC were observed to be enhanced for a particular range of lengths of cut (10, 15, 20 and 25 mm) and volume content of jute yarn (0.1, 0.25, 0.5 and 0.75 %). The maximum increment of compressive, flexural and tensile strengths observed in the investigation are 33, 23 and 38 %, respectively with respect to concrete without jute yarn.

## Background

The necessity of sustainable, non-hazardous and cost effective materials in the construction industry is inevitable. Concrete being popularly used all over the world is characterised by low strength-strain capacity in tension, and low fracture toughness. To overcome the shortcomings of plain concrete (PC), the use of reinforcing fibre has been found very effective (Thakur et al. 2014; Balaguru and Shah 1992; Barr et al. 1996; Meddaha and Bencheikh 2009; Rizkalla and Hassan 2002). The fibres can insure the post-cracking resistance, high-energy absorption features and increased fatigue resistance of cement based composites (Savastano et al. 2009). Among two different types of fibres i.e., natural fibres and artificial polymer based fibres, natural fibres are promising to use as reinforcement to overcome the inherent deficiencies in FRCC reinforced with polymer based fibre (Ramakrishna and Sundararajan 2005). The main deficiencies associated with the use of artificial fibres are: relatively high cost, health and environmental hazards.

On the contrary, natural fibres which are biodegradable, inexpensive, environmental friendly, easy availability as reported by (Xie et al. 2010) are produced from naturally available resources for instance, coconut tree, banana tree, cotton, jute, etc. Researchers have conducted numerous studies on the effect of natural fibres on the mechanical and physical behaviour of concrete to investigate the extent of improvement. In recent years, unrelenting efforts has been observed for using natural fibres in FRCC for improving the energy-efficiency, economy and eco-friendliness flavor (Ramakrishna and Sundararajan 2005). Hence, the demands to utilise natural fibres for making good quality and low-cost, sustainable FRCC for housing and other necessities are increasing. Additionally, the other potential application of natural fibre reinforced cement composites are limited to those area where energy are to be absorbed or the areas prone to impact damage. Accordingly, natural fibre reinforced cement composites are most suitable for shatter and earthquake resistant construction, foundation floor for machinery in factories, fabrication of light weight cement based roofing and ceiling boards, wall plaster, and construction materials for low cost housing (Aziz et al. 1981). Variety of factors influences mechanical properties of FRCC reinforced with natural fibre. The factors are: characteristics of fibres, nature of the cement based matrix, and the way of mixing, casting and curing the composite (Aziz et al. 1981). Among these parameters, the type of fibre and their characteristics have a significant influence on the mechanical properties of these composites (Jarabo et al. 2012). Jute fibres, which come from annual plants, are available in plenty in Bangladesh. It is a prospective material for cement based matrix.

According to the previous study (Mansur and Aziz 1982; Chakraborty 2013; Meddaha and Bencheikh 2009; Bezerra et al. 2004) a number of difficulties are encountered while mixing natural fibres to produce composite. For instance, Meddaha and Bencheikh (2009) mentioned that inhomogeneous distribution of fibres yields bulk and surface flaws. The stress concentration at these flaws would accelerate crack propagation which results lower fracture strength of the mortar specimens (Bezerra et al. 2004). Chakraborty (2013) also reported to use natural fibres in FRCC that the agglomeration could not be avoided. The amount of fibres that can be added to a mix is limited by the tendency of ‘bailing’ (Mansur and Aziz 1982) where the fibres frequently intermesh and form fibre balls which is critical to be separated. The bailing of fibres results in an ineffectual and segregated mix which produces a highly porous and honeycombed concrete. As a result, a remarkable strength fall of the concrete composite occurs. From the above elucidation, it can be concluded that the fibre mixing plays a vital role in the perfection of mechanical properties of fibre reinforced concrete composites. On the other hand, jute yarn processing covers a long process line during its manufacturing along with fibre opening, cleaning, faults removing and parallelizing (Muttaki 2013). Furthermore, the loose dust is eliminated by softener where dirt falls off and pieces of bark and stick become damaged from the jute fibre during the process (Atkinson 1985). Carding machine initiates the parallelizing of the jute fibre and eventually drafting and doubling section also adds value to it (Atkinson 1985). However, the effect of using jute yarn on the mechanical behaviour of concrete reinforced with jute yarn has yet to be investigated. Additionally, the use natural fibres in concrete made with crushed stone have been universally used. The use of crushed bricks are very common (Rashid et al. 2009) in Bangladesh, parts of India and some other countries having scarcity of stone. Additionally, the mechanical properties of concrete made with crushed bricks are quite different than those made with crushed stone (Mansur et al. 1999; Mohammed et al. 2015). Investigative studies have not yet been conducted to evaluate the effect jute fibre or jute yarn on the concrete made with crushed bricks. To this end, it is very much rationale to determine the effect for exploring potential use of jute for obtaining enhanced concrete with low cost, without affecting the environment and minimum health hazard. With this background, the main objective of the study is to develop jute yarn-reinforced concrete composites and to investigate the effect of yarn length and content (volume fraction) on its mechanical behaviour.

## Methods

### Materials

**Fig. 1:**
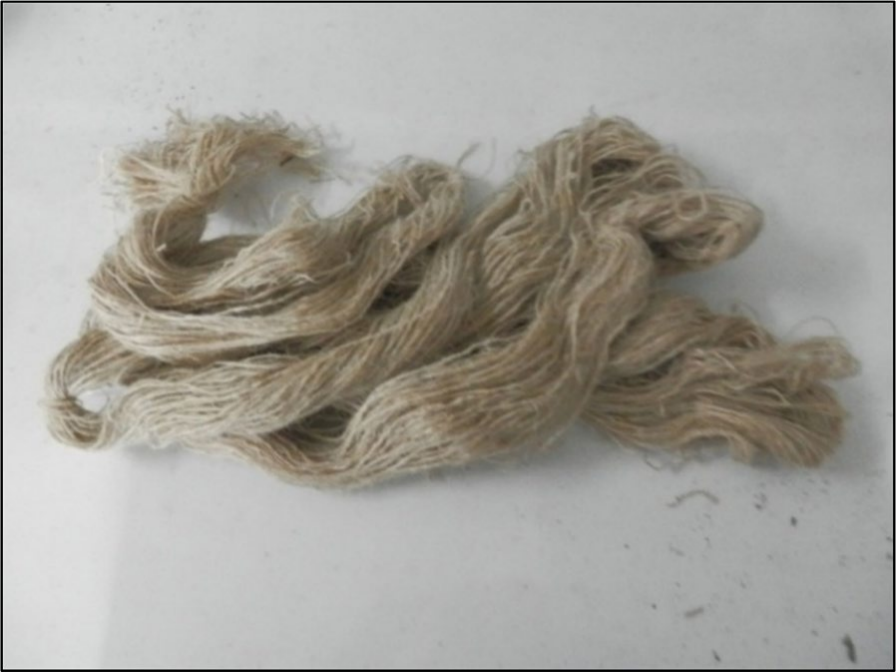
Raw jute yarn

**Fig. 2:**
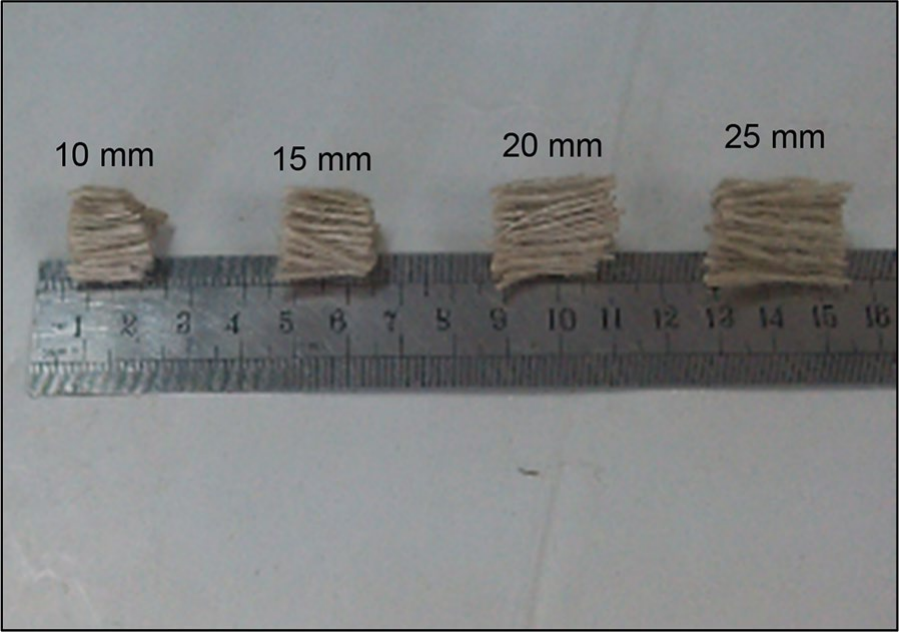
Jute yarn of different cut lengths

The locally available raw jute yarn of 10 lb/spyndle with 5 TPI (Twist per inch) shown in Fig. 1, was used without any treatment. This jute yarn with four different cut lengths (10, 15, 20 and 25 mm) also shown in Fig. 2, were applied with various volumetric percentage on concrete mixture. Ordinary Portland cement were applied as binding materials, normal consistency of which was 30 %, initial setting time was 132 min and the final setting time was 7.00 h. Sand (fineness modulus = 2.5) and 25 mm down well graded crushed bricks were used as coarse aggregate.

### Concrete mix

Mix design is the selection of mix ingredients and their proportions required in a concrete mix. The mix design involves that amount of cement, fine aggregate, and coarse aggregate be available and the relation between water/cement ratio and target strength must be known. Since, the objective of the study is to investigate the effect of incorporating jute yarn on the mechanical properties of concrete, the mix design with target strength was not accomplished in the study. Rather, the commonly practised mix ratio used in Bangladesh and other neighbouring countries like parts of India and Pakistan was used. To this end, in the present study, two different mix ratios, cement: sand: brick chips (by volume) = 1:2:4 and 1:1.5:3 and water/cement ratio (by weight) 0.60 and 0.55 were cautiously maintained. In concrete mix preparation, initially different jute yarn cut length and volumetric content were applied in the concrete mix and observed the mixing performance to obtain better arrangement of jute. And finally jute yarn of 10, 15, 20 and 25 mm lengths with 0, 0.1, 0.25, 0.50 and 0.75 % volume dosing were selected; and lastly samples were prepared for a particular set of parameters.

Table 1 shows the calculated amount of materials for a single variable, where others can be achieved by the same procedure.

**Table 1:** Amount of various materials for preparation of a Prism with mix ratio 1:1.5:3

Sl. no.	% of Fiber or yarn (as volume)	Total volume (cm^3^)	Volume of Cement & Aggregates (cm3)			Volume of Jute(cm^3^)	Specific gravity of jute (gm/cm^3^)	Weight of Jute (gm)
			Cement	Sand	Brick chips			
1	0	10618.82	1930.69	2896.04	5792.08	0.00	1.46	0.00
2	0.1		1928.76	2893.15	5786.29	10.62		15.50
3	0.25		1925.87	2888.80	5777.60	26.55		38.76
4	0.5		1921.04	2881.56	5763.12	53.09		77.52
5	0.75		1911.39	2867.08	5734.16	106.19		155.03

### Preparation of the test specimen

The different parameters of the concrete composites, the length and volume fraction of the jute yarn content were used. The different lengths of yarn 10, 15, 20 and 25 mm and the contents 0, 0.1, 0.25, 0.50 and 0.75 % were used. Three different specimens; cubes (150 mm × 150 mm × 150 mm), prisms (450 mm × 150 mm × 150 mm) and cylinders (150 mm × 300 mm) were cast to determine the compressive, flexural and tensile strength of the composites, respectively. The yarns were cut to the mentioned length manually by a hand scissor. The mixing of the ingredients were done by a pan mixer accordingly jute yarns were added slowly and evenly to the concrete mix, so that, a uniform distribution of yarns throughout of the concrete could be confirmed. Cement was added into the mixer and mixing was conducted followed by the addition of water, until uniformity was achieved. This way of concrete mixing was continued for about 3 min. Then the freshly mixed concrete was poured in the moulds of cube, prism and cylinder. After that the specimens were left 24 h for de-moulding. They were then cured in water for at least 28 days. At the end of curing period the specimens were allowed to be dried in air for 24 h before testing.

### Experimental program

**Fig. 3:**
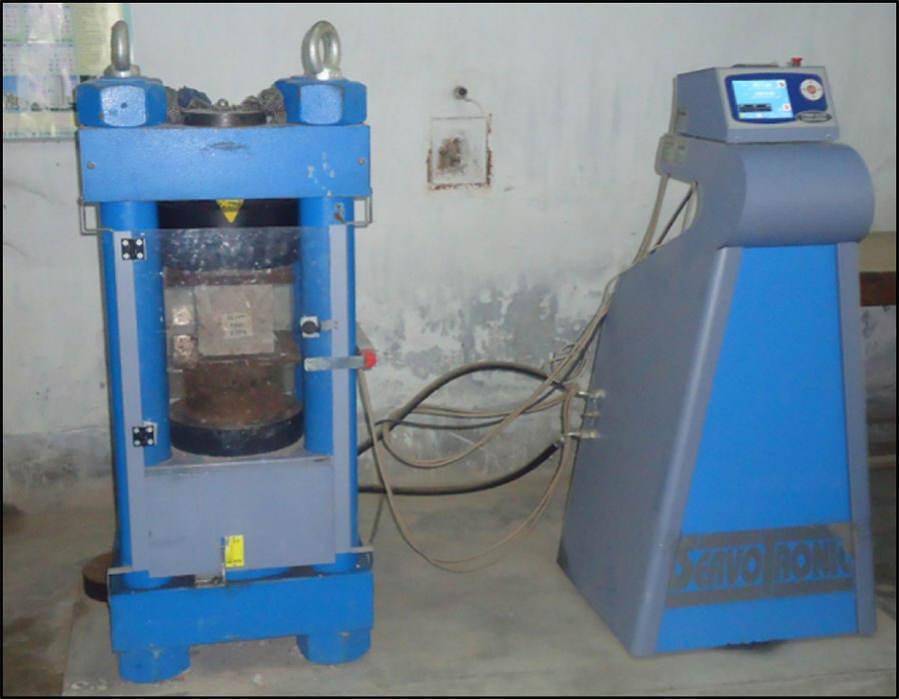
Automatic Compression Testing Machine (MATEST s.r.l.)

The present study consists of determining the flexural, compressive and tensile strength of concrete composites with jute yarn and was compared to that of plain concrete. A Universal Testing Machine (Model-UTN-100, India, Capacity-980 KN.) for tensile test and an Automatic Compression Testing Machine (MATEST s.r.l, Italy, capacity 3000 KN) shown in Fig. 3, for compressive test and an Automatic Flexural Strength Testing Machine (MATEST s.r.l, Italy, Capacity 150 KN.) for flexural test were used. In addition, the microscopic views of tested specimens were also analysed.

### Compressive strength testing

Compressive strength of a concrete is a measure of its ability to resist static load, when the later one tends to crush it. Testing of compressive strength is the most common, many desirable characteristics of concrete are related to its strength, and hence the compressive strength of concrete in structural design is of utmost importance. Additionally, the compressive strength gives a good and clear indication that how the strength is affected with the increase of fibre volume dosage rate in the test specimens. In AS 1012, it is mentioned that the specimens for compressive strength should be 150 mm diameter and 300 mm height, but this only applies to the maximum aggregate size more than 20 mm while the cube specimen with 150 mm in each side (AS 1012 2002), and the intensity of load is determined in MPa. qualitatively. The compression test procedure was carried out according to test method AS 1012.9.

### Flexural strength testing

Flexural strength of a concrete is a measure of its ability to resist bending and it can be expressed in terms of modulus of rupture. Therefore, the two point loading method was used in making flexural strength tests of concrete employing bearing blocks which ensured that forces applied to the beam was perpendicular to the face of the specimen and was applied without eccentricity. During test the reaction was always parallel to the direction of applied force. The test procedure was carried out following the test method ASTM C 78-00. The distance of the loading point (*l*) is 133 mm and the supporting point (L) is 400 mm whereas the load was applied continuously and without any shock at a constant rate to the breaking point. Apply the load at a rate that constantly increases the extreme fibre stress 1.21 MPa./min. Finally, results were obtained in the form of total load in KN and intensity of load in MPa.

### Tensile strength testing

Investigation of concrete’s mechanical properties can be presented reasonably through the analysis of tensile strength. The brittleness and low tensile strength of concrete make it abortive to struggle with the direct tension. Hence the measurement of tensile strength is obligatory to determine the load at which the concrete members may crack therefore the cracking is due to the tension failure. The splitting tests (sometimes referred to as split tensile strength tests) are well known indirect tests used for determining the tensile strength of concrete. The test procedure consists of applying a compressive line load along the opposite generators of a concrete cylinder placed with its axis horizontal between the compressive planes. The splitting tensile strength test was conducted according to the test method ASTM C 496/M496.

## Results and discussions

**Fig. 4:**
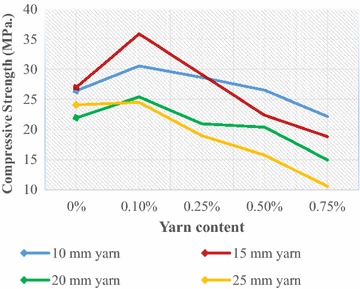
Compressive strength of JYRCC with 1:1.5:3 mix ratio

**Fig. 5:**
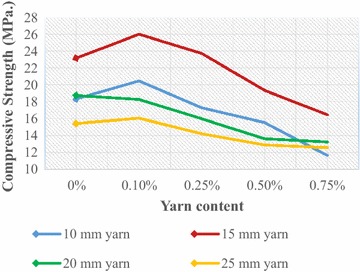
Compressive strength of JYRCC with 1:2:4 mix ratio

**Fig. 6:**
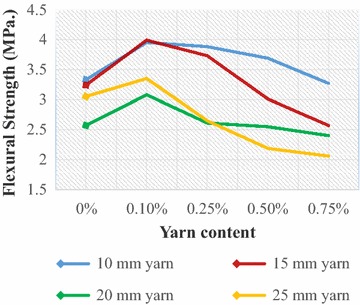
Flexural strength of JYRCC with 1:1.5:3 mix ratio

**Fig. 7:**
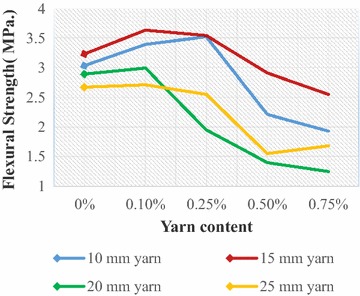
Flexural strength of JYRCC with 1:2:4 mix ratio

**Fig. 8:**
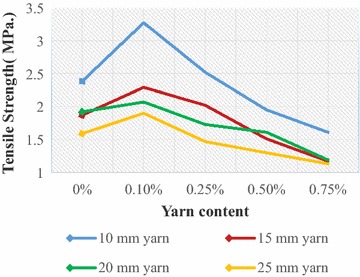
Tensile strength of JYRCC with 1:1.5:3 mix ratio

**Fig. 9:**
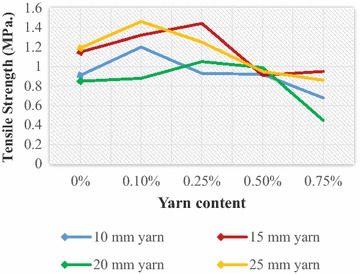
Tensile strength of JYRCC with 1:2:4 mix ratio

The mechanical behaviour of fibre-cement composite, which basically accounts for the bond between fibre and surrounding concrete, largely depends on many factors like the physical characteristics of the fibres such as geometry, type and surface characteristics, fibre orientation, fibre volume ratio and fibre distribution, the chemical composition of the fibre and so on. Tests were conducted to evaluate the effect of jute yarn and results are presented diagrammatically in figures (Figs. 4, 5, 6, 7, 8, 9).

### Compressive strength

Figure 4 represents the variation of compressive strength with respect to yarn content and yarn length for concrete mix ratio 1:1.5:3. A particular curve represents the variation of the compressive strength with volumetric content of yarn. It is to mention that 0 % yarn content represents that no yarn was mixed which indicates plain concrete. From the figure no specific trend with yarn length with yarn content can be observed. Compressive strength increment up to a certain yarn content that is 0.1 % with yarn lengths can be seen for 10, 15, and 20 mm yarn while that is found to decrease for 25 mm yarn. The rate of increase for 15 mm yarn with 0.1 % content is the largest. With further increase of yarn content the compressive strength is seen to decrease for different lengths of yarn. The reason for irregular variation can be interpreted in the following way. In the case of larger yarn length (yarn length >15 mm) and content (>0.25 %) difficulties were encountered to maintain consistency in concrete mix. Since numerous studies have considered reinforcing materials i.e. fibres or yarns as similar to coarse aggregate, the inclusion of jute yarn leads to an increase of coarse aggregate fraction in spite of the fine aggregate fraction which could result in a high porosity in the cement matrix. For decreasing trend with yarn content can be explained that with the addition of jute yarn in concrete reduces the specific gravity of the composites and due to the low specific gravity, inadequate mixing and high porosity of the JYRCC, a lower compressive strength with respect to the reference concrete particularly, when a high volume and larger length of yarn was added. Similar results were obtained by Shimizu and Jorillo (1992). However, the short yarn length with smaller content, which act as short jute fibre was firmly bound the composite constituent, and develop an intact material which results the more resistance to applied force. Similar trends can be observed in Fig. 5 which represents the results for concrete mix ratio 1:2:4. In Fig. 5, the maximum compressive strength increment can be seen for 15 mm yarn and 0.1 % yarn content. Similar complexities in making concrete composites with different fibre length and content have been reported by Barr et al. (1996). The maximum improvement of compressive strength was found for 15 mm yarn length and 0.10 % content both 1:1.5:3 and 1:2:4 mix ratio. The improvements are 33 % and 12 % for 1:1.5:3 and 1:2:4 mix ratio with respect to plain concrete, where fine distribution of reinforcing materials i.e. jute yarn are obtained. Finally, it can be said that JYRCC with <15 mm yarn cut length with ≤0.10 % volumetric dozing is the more promising combination for compressive strength increment.

**Fig. 10:**
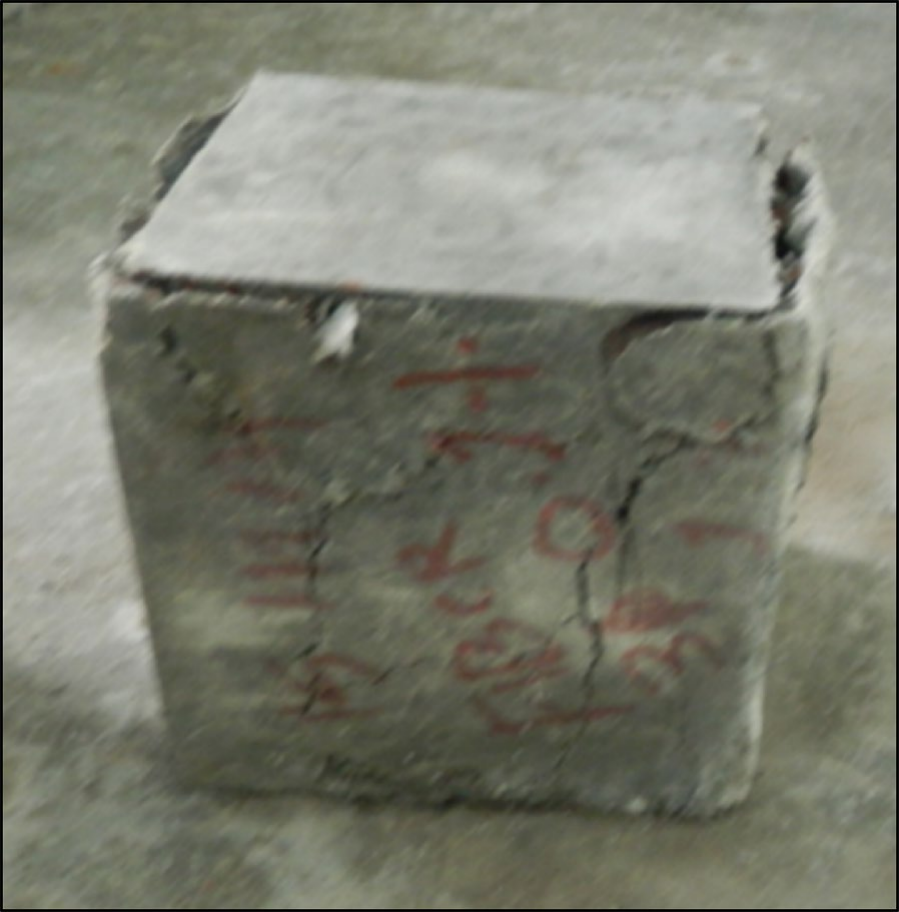
JYRCC specimen after compression failure

**Fig. 11:**
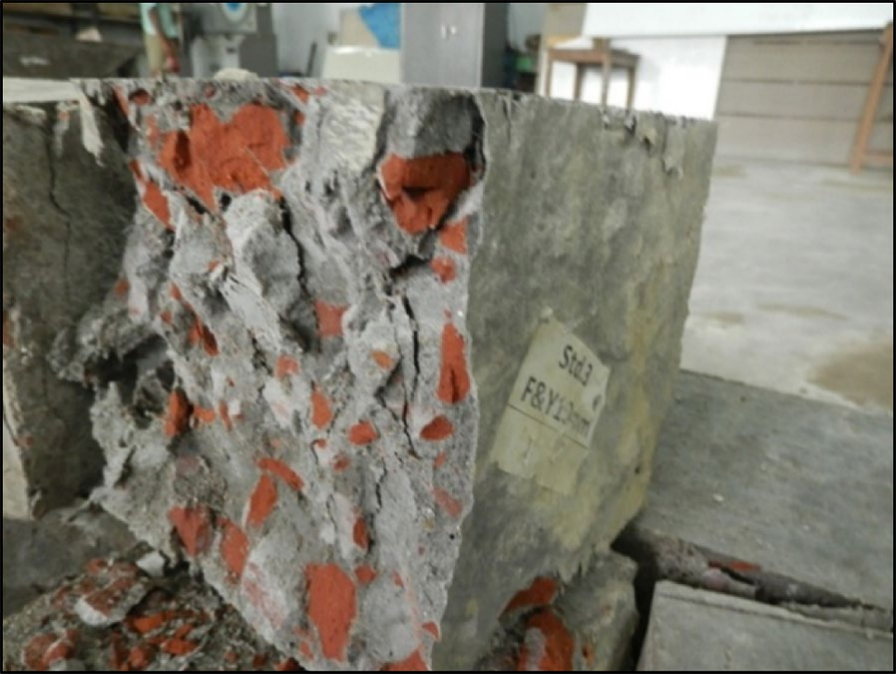
Control specimen after compression failure

Figures 10 and 11 show the failure specimens of the sample after tests. In the case of control specimen, it was seen that the crack propagates rapidly with a regular manner while the cracks were observed to run in multidirectional path for JYRCC. This is possible through stress transfer across the cracks and the fibre arrests the rapid crack propagation and prolongs the strain life to continue beyond the ultimate.

### Flexural strength

Figures 6 and 7 illustrate the flexural strength of JYRCC with different jute yarn lengths (10, 15, 20 and 25 mm) and yarn content (0, 0.1, .25, 0.5 and 0.75 %) for 1:1.5:3 and 1:2:4 concrete mix ratio respectively, where 0 % yarn content represents the plain concrete. From Fig. 6 significant influence on the flexural strength for yarn length 10 mm and 15 mm with volumetric content from 0.1 to 0.25 % cab be observed. In contrast the flexural strength fall can be seen for the further increment of yarn length and content. Almost similar results were obtained for mix ratio 1:2:4 shown in Fig. 7. However, the considerable decrements of strength were found for higher yarn length and content. The increment of yarn length (>15 mm) and yarn volume (>0.25 %) in concrete causes the balling of JYRCC creates high porosity and weak zones in the specimen, therefore, a decrease in flexural strength was observed. Similar trend has also been observed regarding the incorporation of fibre in concrete by (Meddaha and Bencheikh 2009). Failure of such beam specimen, are initiated by formation of cracks that would proceed along the pre-weak zones with smaller cracking load as compared with the intact specimen or with specimen with lower yarn content. The matted condition of concrete mixture is irradiate by using low reinforcing material cut length and jute spun yarn as reinforcing material. During mixing of concrete low cut length’s jute yarn de-twist slowly and causes the even spreading of fibre and create the better reinforcement on composites. Finally the largest value of flexural strength augmentation was achieved 23 % for 1:1.5:3 mix ratio with 15 mm yarn length of 0.10 % volume dozes and 16 % for 1:2:4 mix ratio with 10 mm yarn length of 0.25 % yarn content

### Tensile strength

The variation of tensile strength with respect to yarn content (0, 0.1, 0.25, 0.5 and 0.75 %) and yarn lengths (10, 15, 20 and 25 mm) are shown in Figs. 8 and 9, for concrete mix ratio 1:1.5:3 and 1:2:4, respectively. Larger fibre content yields more voids in the concrete due to the lack of free reorganization of the concrete matrix as a result of the reduced workability and balling effect during vibration and casting of the specimens. Schrader (1978) mentioned that the first crack load of FRC depends basically on the amount, length configuration, strength and ductility of fibres, whereas cement content and aggregates are less responsible in this concern. The contribution of the jute fibre is observed on the ability of JFRC composite to maintain the ultimate load through further deflection without sudden collapse. Figures 8 and 9 express the increasing trend of tensile strength with yarn content 0.1 and 0.25 % for 10 and 15 mm yarn length.

### Analysis the microscopic view of JYRCC

**Fig. 12:**
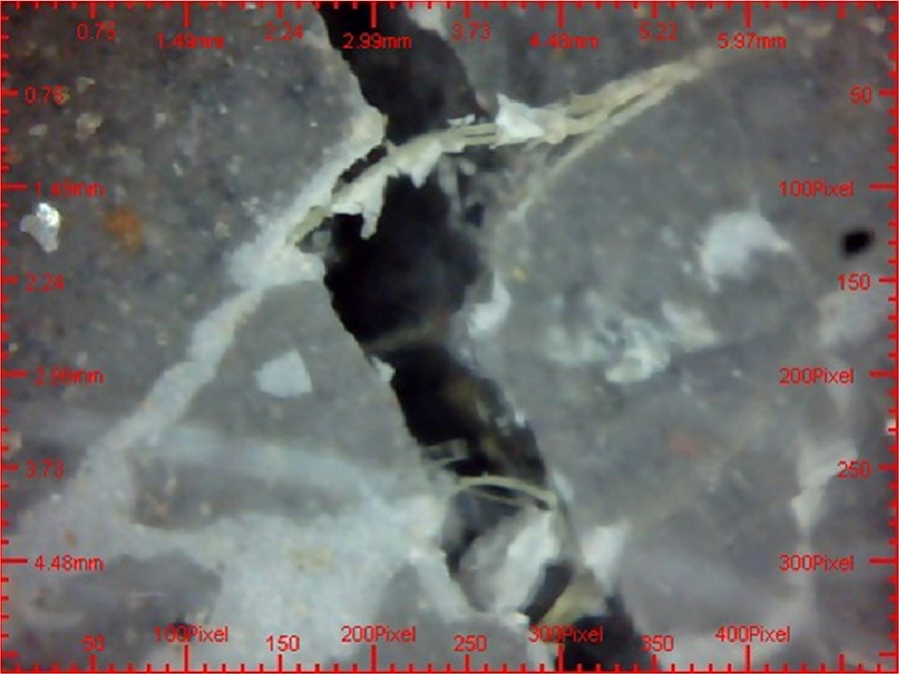
Microscopic image (X50) of crashed JYRCC after compression test

**Fig. 13:**
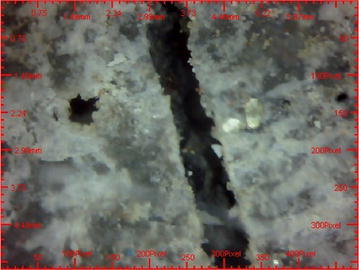
Microscopic image (X50) of crashed plain concrete after compression test

Microscopic images of failed JYRCC specimen were taken and shown in Figs. 12 and 13. From the microscopic view, visual analyses were done. Images taken on crack parts of sample shows the presence of randomly distributed jute fibre which caused the increment of strength of JYRCC. Fractured ends of jute fibre were appeared in the specimen that showed the largest strength enhancement. From the observation it would be stated that due to the adequate bonding between jute fibre and concrete causes the fibre breakage at the cracked line that’s why JYRCC expressed the strength augmentation with reference to plain concrete. It can also be visualized that the jute fibre resists crack in different angle and brace it that is absent in plain concrete. For this reason at the time of failure of various test the JYRCC specimen part is not discrete properly but for plain concrete it would be separate very quickly, it may convey the better results for developed earthquake resistive structure.

## Conclusion

In the experimental investigations conducted in the study, it was found that, the addition of jute yarn contributes enriched results for mechanical properties of concrete composites for a particular yarn length and yarn content. More specifically, compressive, flexural, and tensile strength are found to enhance significantly for volume content of 0.1 and 0.25 % and the yarn cut length of 10 and 15 mm. However, with larger yarn length and content the mechanical properties were found to affect adversely. Finally the jute yarn shows the positive contribution to minimize irregular mixing disabilities of concrete composites that creates major problem regarding fibre reinforcement. So JYRCC can be developed with locally fabricated jute yarn in Bangladesh. The least cost of jute yarn, its being renewable resources, the reduced weight of the JYRCC and the environmental compatibility would clearly show the socioeconomic viability of JYRCC. This diversified use of jute may redeem the lost glory of jute in Bangladesh.

## References

[CR1] AS 1012 (2002) Compressive test of concrete specimen, Methods of testing concrete, Standards Australia

[CR2] Atkinson RR (1985). Jute Fibre to yarn.

[CR3] Aziz MA, Paramasivam P, Lee SL (1981). Prospects for natural fibre reinforced. Int J Cem Comp Lightweight Concr.

[CR4] Balaguru P, Shah S (1992). Fibre-reinforced cement composites.

[CR5] Barr B, Gettu R, Al-Oraimi S, Bryars L (1996). Toughness measurement—the need to think again. Cem Concr Comp.

[CR6] Bezerra E, Joaquim A, Savastano H Jr (2004) Some properties of fibre–cement. In: NOCMAT conference 2004. Pirassununga, Brasil, pp 1–11

[CR7] Chakraborty S, Kundu SP, Roy A, Basak RK, Adhikari B, Majumder SB (2013). Improvement of the mechanical properties of jute fibre reinforced cement mortar: a statistical approach. Constr Build Mat.

[CR8] Jarabo R, Fuente E, Monte MC, Savastano H, Mutjé P, Negro C (2012). Use of cellulose fibers from hemp core in fiber-cement production. Effect on flocculation, retention, drainage and product properties. Ind Crops Prod.

[CR9] Mansur M, Aziz M (1982). A study of jute fibre reinforced cement composites. Int J Cem Comp Lightweight Concr.

[CR10] Mansur MA, Wee TH, Cheran LS (1999). Crushed bricks as coarse aggregate for concrete. ACI Mat J.

[CR11] Meddaha MS, Bencheikh M (2009). Properties of concrete reinforced with different kinds of industrial waste fibre materials. Constr Build Mat.

[CR12] Mohammed TU, Ariful Hasnat S, Awal MA, Bosunia SZ (2015) Recycling of brick aggregate concrete as coarse aggregate. J Mat Civil Eng 27(7)

[CR13] Muttaki S (2013) A comprehensive study on jute fiber. 2nd ed. s.l.: Textile Bulletin

[CR14] Ramakrishna G, Sundararajan T (2005). Impact strength of a few natural fibre reinforced cement mortar. Cem Concr Comp.

[CR15] Rashid MA, Hossain T, Islam MA (2009). Properties of higher strength concrete made with crushed brick as coarse aggregate. J Civil Eng (IEB).

[CR16] Rizkalla S, Hassan T (2002). Effectiveness of FRP for strengthening concrete bridges. Struct Eng Int.

[CR17] Savastano H, Santos S, Radonjic M, Soboyejo W (2009). Fracture and fatigue of natural fiber-reinforced cementitious composites. Cem Concr Comp.

[CR18] Schrader EK, Swamy RN (1978). Formulating guidance for testing of fibre concrete in ACI Committee 544. Testing and test methods of fibre cement composites.

[CR19] Shimizu G, Jorillo PJ (1992) Coir fibre reinforced cement based composite. Part one: microstructure and properties of fibre-mortar. In: London, Proceedings 4th RILEM international symposium on fibre reinforced cement and concrete, pp 1080–1095

[CR20] Thakur VK, Thakur MK, Gupta RK (2014) Graft copolymers of natural fibers for green composites. Carbohydr Polym 104:87–93 10.1016/j.carbpol.2014.01.016 24607164

[CR21] Xie YJ, Hill CA, Xiao Z, Militz H, Mai C (2010). Silane coupling agents used for natural fiber polymer composites: a review. Comp Part A Appl Sci Manuf.

